# Pathways of physical activity behavior after an intervention with students from vulnerable areas: a cluster randomized controlled trial based on a socioecological approach

**DOI:** 10.1590/0102-311XEN138023

**Published:** 2024-11-11

**Authors:** Jaqueline Aragoni da Silva, Valter Cordeiro Barbosa, Alexsandra da Silva Bandeira, Kelly Samara da Silva, Jorge Mota

**Affiliations:** 1 Universidade Federal de Santa Catarina, Florianópolis, Brasil.; 2 Universidade Federal do Ceará, Fortaleza, Brasil.; 3 Faculdade de Desporto, Universidade do Porto, Porto, Portugal.

**Keywords:** Physical Activity, Health Education, Social Vulnerability, Atividade Física, Educação em Saúde, Vulnerabilidade Social, Actividad Física, Educación en Salud, Vulnerabilidad Social

## Abstract

Efforts are needed to better understand what are the effective pathways that can optimize success in school-based physical activity interventions. This study aimed to investigate the mediators of a school-based intervention in the practice of physical activity in Brazilian students. The *Fortaleça sua Saúde* [Strengthen Your Health] program followed 1,085 students (11-18 years) over a semester. This multi-component intervention included training teachers, offering physical activity opportunities, and health education. Self-reported moderate-to-vigorous physical activity and potential mediators (attitude, self-efficacy, social support, perceived neighborhood environment, and physical activity facilities at school) were assessed. The product of coefficient analysis was performed. The sample was composed of 1,085 students (51.5% boys). The total effect of the intervention was 0.706 (95%CI: 0.276; 1.136). A total of 40% of the intervention effect on moderate-to-vigorous physical activity was explained by attitude towards physical activity and social support from friends and teachers. Social support from friends was a significant mediator only among boys (*ab*: 0.113, 95%CI: 0.027; 0.256), and social support from teachers only among girls (*ab*: 0.135, 95%CI: 0.019; 0.328); indicating a statistically significant indirect effect of the program on moderate-to-vigorous physical activity via these mediators. A relevant part of the effect of a multicomponent intervention on physical activity among students from vulnerable areas is explained by changes via variables at different levels of the socioecological model, including social support from friends and teachers, and attitude towards physical activity. These results should be considered in public policies.

## Abstract (pt)

São necessárias esforços para melhor entender quais são os caminhos efetivos que podem otimizar o sucesso em intervenções de atividade física escolares. Este estudo investigou os mediadores de uma intervenção escolar na prática de atividade física entre estudantes brasileiros. O programa Fortaleça sua Saúde matriculou 1.085 alunos (11-18 anos) ao longo de um semestre. Essa intervenção multicomponente incluiu a capacitação de professores, a oferta de oportunidades de atividade física e a educação em saúde. Atividade física moderada a vigorosa autorrelatada e potenciais mediadores (atitude, autoeficácia, suporte social, ambiente da vizinhança percebido e instalações de atividade física na escola) foram avaliados. Foi realizada a análise do produto do coeficiente. A amostra foi composta por 1.085 estudantes (51,5% meninos). O efeito total da intervenção foi de 0,706 (IC95%: 0,276; 1,136). No total, 40% do efeito da intervenção sobre atividade física moderada a vigorosa foi explicado pela atitude em relação à atividade física dos participantes e apoio social de amigos e professores. O apoio social dos amigos foi um mediador significativo apenas entre os meninos (*ab*: 0,113, IC95%: 0,027; 0,256) e apoio social dos professores apenas entre as meninas (*ab*: 0,135, IC95%: 0,019; 0,328), indicando um efeito indireto estatisticamente significativo do programa sobre a atividade física moderada a vigorosa por meio destes mediadores. Parte relevante do efeito de uma intervenção multicomponente sobre atividade física entre estudantes de regiões vulneráveis é explicada por mudanças por meio de variáveis em diferentes níveis do modelo socioecológico, incluindo apoio social de amigos e professores e atitude em relação à atividade física. Estes resultados devem ser considerados nas políticas públicas.

## Abstract (es)

Se necesitan esfuerzos para comprender mejor cuáles son las opciones efectivas para optimizar el éxito en las intervenciones de actividad física escolares. Este estudio investigó los mediadores de una intervención escolar en la práctica de actividad física entre estudiantes brasileños. El programa *Fortaleça sua Saúde* [Fortalece tu Salud] inscribió a 1.085 estudiantes (11-18 años) a lo largo de un semestre. Esta intervención multicomponente incluyó la capacitación docente, la oferta de oportunidades de actividad física y la educación en salud. Se evaluaron la actividad física de moderada a vigorosa autoinformada y los potenciales mediadores (actitud, autoeficacia, apoyo social, entorno residencial percibido e instalaciones de actividad física en la escuela). Se realizó el análisis del producto del coeficiente. La muestra estuvo compuesta por 1.085 estudiantes (51,5% niños). El efecto total de la intervención fue de 0,706 (IC95%: 0,276; 1,136). En total, el 40% del efecto de la intervención sobre actividad física de moderada a vigorosa se explicó por la actitud de los participantes con relación a la actividad física y el apoyo social de amigos y profesores. El apoyo social de los amigos fue un mediador significativo solo entre los niños (*ab*: 0,113, IC95%: 0,027; 0,256) y el apoyo social de los docentes solo entre las niñas (*ab*: 0,135, IC95%: 0,019; 0,328), lo que indica un efecto indirecto estadísticamente significativo del programa sobre la actividad física de moderada a vigorosa a través de estos mediadores. Una parte relevante del efecto de una intervención multicomponente sobre la actividad física entre estudiantes de regiones vulnerables se explica por cambios a través de variables en diferentes niveles del modelo socioecológico, incluido el apoyo social de amigos y profesores y la actitud con relación a la actividad física. Estos resultados deben tenerse en cuenta en las políticas públicas.

## Introduction

Schools have been recognized as potential contexts that can play an important role in societies and people’s life. The primary role of schools is to provide children with educational opportunities that include academic subjects and a range of life skills essential for health and social development. Due to the strong relation between physical activity behaviors (e.g., active commuting to schools, playing games, and other movement behaviors during and after school time) and global health, physical activity should be included in the subjects addressed in the school context ^1^ ^,^ ^2^. Moreover, Physical Education classes can positively impact not only physical health but also affective, cognitive, and social aspects ^3^. Health literacy, an important aspect for promoting long-term healthy and active lifestyles, can be facilitated via quality Physical Education classes ^1^.

Behavior change is a complex process in which the aforementioned factors depict only a fraction of what can influence an individual’s behavior. Different theory-based models aim to explain physical activity, including sociocognitive, humanistic, and dual-process models. A common characteristic of all models is their focus on the individual level ^4^, differing from the socioecological model that postulates the existence of different levels of influence in physical activity practice due to its multidimensionality ^5^. For instance, the model suggests that physical activity behavior can be modified by factors from different levels, including individual (self-efficacy, perceived risk, and benefit), interpersonal (social support), and environmental (self-perception of school and neighborhood physical activity context) levels ^2^.

Therefore, evidence supports that strategies that consider the whole school approach (from teaching to the physical and social school environment, as well as individual behavior factors) can impact students’ health and physical activity ^2^. Multicomponent approaches that consider the different aspects of the school environment are the most commonly employed type of intervention, with the strongest evidence that it can positively impact physical activity and its potential determinants6.

Although several efforts have been made so far, many aspects still need to be improved, as evidence shows that the effects of school-based interventions are still generally null or small; optimal models of school-based interventions need yet to be elucidated ^6^ ^,^ ^7^. Moreover, most of the evidence on promoting physical activity in young people comes from high-income countries ^6^. An overview on this topic concluded that only 3.1% of the studies mentioned in systematic reviews were from low- and middle-income countries ^8^. People in these contexts have the greatest exposure to health-related risk factors and violence, and restricted access to education and healthy lifestyles compared to those in rich countries ^9^. These factors can adversely impact the population’s health behavior ^10^. The evaluation of physical activity interventions in vulnerable regions is highly urgent, as context-specific evidence can aid to better target the population according to their particular needs.

A potential way to overcome these gaps in the literature is by investigating their mediating mechanisms (i.e., the variables in the pathways between an intervention and physical activity behavior) ^11^. Reviews on this topic ^11^ ^,^ ^12^ ^,^ ^13^ have highlighted that most studies failed to consider individual and contextual physical activity factors (e.g., social support and self-efficacy) in the intervention design and evaluate the mechanisms of the physical activity behavior change via these factors. Moreover, researchers often carry out cross-sectional mediation models due to their feasibility for studies with limited resources, although causality cannot be assumed due to the lack of temporality between measurement points. Nonetheless, these models can contribute to the literature and pave the way for the formulation of new hypotheses to be tested by studies that can afford the complexity of data collection in multiple time points ^14^. Understanding which factors are mediators of interventions can aid design the critical components of the programs and policies on physical activity ^15^. This will allow researchers and policymakers to draw more reliable and comprehensive physical activity promotion.

Another challenge regarding promoting physical activity is that gender-related inequality in physical activity participation is still a current concern ^1^. Recently, minimal progress has been made in mitigating sex-based differences in physical activity ^16^. Thus, places such as schools must be supportive and equitable to reverse disparities in physical activity ^1^. In these contexts, the need to provide equitable physical activity opportunities ^17^ and separately investigate gender disparities in physical activity patterns have been highlighted ^6^. Then, public policies can advance towards a more inclusive and comprehensive approach to promoting physical activity that considers and rectifies gender-related imbalances.

Therefore, this study aims to investigate the intrapersonal, interpersonal, and environmental mediators of a school-based, multicomponent program aimed at promoting physical activity in boys and girls from areas with low Human Development Index (HDI).

## Material and methods

This study follows the Consolidated Standards of Reporting Trials (CONSORT) ^18^. The *Fortaleça Sua Saúde* (Strengthen Your Health) is a school-based cluster randomized controlled trial aimed at increasing physical activity engagement and reducing screen time of students aged 11-18 years old. The study was conducted in Fortaleza, Ceará State, Brazil, over a semester in 2014 (recruitment: July 2014; follow-up: December 2014, ending due to the school year calendar) in an area with a low HDI (0.170-0.491). Schools were eligible if they showed a full-time system and had incorporated the Brazilian School Health Program (a national program to promote health in schools). All six eligible schools were included and randomly assigned to the control (CG; n = 3) or intervention (IG; n = 3) group. Students in the 7th to 9th grades were eligible to take part in the study, and 1,085 (85%) provided baseline and follow-up measurements. Losses and exclusions were mainly due to refusals (CG = 3, IG = 1), absences (CG = 35, IG = 47), and missing data (CG = 1, IG = 3). Participant flowchart is available elsewhere ^(19)^. Authorization to participate in the study was obtained from their guardians, who signed an informed consent form. The study was approved by the Brazilian National Research Ethics Comission (protocol n. 17366313.9.0000.0121). A detailed description of the selection process and sample characteristics can be found in previous publications ^19^ ^,^ ^20^. Information on pre- and post-intervention characteristics can also be found elsewhere ^20^ ^,^ ^21^.

The intervention was based on the socioecological theory and the health promoting schools framework; a detailed description was published elsewhere ^19^. In short, the intervention components and strategies were defined based on the assumption that physical activity is influenced by several levels and their interaction, including individual, interpersonal, and environmental aspects. Moreover, an intervention based on the health promoting schools framework needs to be built by changing all components (i.e., curriculum, social and physical environment of the school, and community/family involvement) that impact the student’s life and health ^2^. Thus, intervention strategies were organized into four main components. All strategies included in these components were designed to influence intrapersonal, interpersonal, and/or environmental variables, as detailed previously ^2^.

The first component was teacher training, in which teachers from the intervention schools participated in face-to-face training and performed classroom lessons that discussed active and healthy lifestyles. A four-hour training session was conducted at the beginning of the school semester addressing the relationship between health, school, and academic performance. Teachers received a supplementary handbook with suggestions for implementing activities into the curriculum. Generally, the classroom activities included the elaboration of texts, videos, posters, and/or booklets (newsletters or flyers) on different health issues, as well as the presentation of these materials to the school community.

A four-hour training was provided to the Physical Education teachers, supplemented by a handbook with suggestions for physical education lessons. Teachers were instructed to teach physical education classes mostly made up of active lessons. Moreover, the Physical Education teachers had the support of an undergraduate Physical Education student during their classes. Extra physical education events were promoted (e.g., games, competitions) aiming to connect physical education with the school cultural agenda. The third component included new opportunities for physical activity practice (social and contextual support for physical activity). They included classes offered by the staff via Gym in School sessions conducted twice per week during break times at school; girls were the target group for these activities. Moreover, new facilities, sports materials, and games were made available to promote physical activity practice. Space and equipment were structured and made available for playing games during free time at school day. All games were supplemented with banners displayed next to them to explain the rules and how to access the equipment needed to play the game.

The last component was focused on health education. It included the production of materials by students (e.g., posters, newsletters, and flyers on health issues), which were available in schools. Posters and newsletters were distributed to the school community and parents. The main topics were related to physical activity, sedentary behavior, and general health. Previously published findings showed that the strategies were satisfactorily implemented - 94% of the teachers implemented health topics in their classes, and half of the students engaged in the proposed activities. A more detailed description is available elsewhere ^22^. Schools in the control group underwent regular activities during the semester ^19^.

Data collection was performed before and after the intervention by the research staff in each school. A training session was provided to the researchers to standardize the administration of the questionnaire. This was conducted based on a previously validated list of 24 types of physical activity (reliability - intraclass correlation coefficient [ICC]: 0.75), including collective physical activity/sports (e.g., volleyball), individual physical activity/sports (e.g., martial arts), ride in physical activity (e.g., skateboarding and cycling), walking, popular games, and other physical activity. Students reported the frequency and duration of physical activity performed in the previous week ^23^. Thus, the main outcome, total moderate-to-vigorous physical activity (minutes/week), was estimated.

Considering previous evidence ^11^ ^,^ ^13^, the potential mediators investigated at pre- and post-intervention were intrapersonal (reliability - Cronbach’s alpha range: 0.77-0.81), interpersonal (reliability - Cronbach’s alpha range: 0.84-0.90), and environmental (reliability - Cronbach’s alpha range: 0.61-0.78). In the context of physical activity, the following aspects were considered: attitude (“Practicing physical activity most days of the week is...”; five items; scores from 5 to 20; the higher, the better), self-efficacy (“I think I can practice physical activity most days of the week even if...”; eight items; scores from 8 to 32; the higher, the better), social support from friends (“How often do your friends:”; five items; scores from 5 to 20; the higher, the better), social support from parents (“How often do your parents:”; six items; scores from 6 to 24; the higher, the better), and social support from teachers (“How often do your teachers:”; five items; scores from 5 to 20; the higher, the better), and perception of school (“At the school where I study…”; three items; scores from 3 to 12; the higher, the better) and neighborhood environment (“In the neighborhood where I live...”; 10 items; subscale scores from 5 to 20; the higher, the better). Detailed information can be found in the Supplementary Material (Table S1; https://cadernos.ensp.fiocruz.br/static//arquivo/supl-e00138023_3745.pdf), and a more detailed explanation of each assessment can be found elsewhere ^13^.

Sociodemographic variables (sex, age, and socioeconomic levels) were obtained using questionnaires. In addition to the contextual socioeconomic level indicator (HDI) of the sample, the characterization of socioeconomic level at the individual level was conducted based on socioeconomic class indicators from the Brazilian Association of Research Companies (ABEP, acronym in Portuguese) ^24^. Based on the reports about the ownership of certain items in the household, parents’ schooling, and number of household employees, adolescents were classified into socioeconomic classes (A to E; from the highest to the lowest level, respectively). In this study, adolescents were grouped into lower (C, D, or E) and higher (A or B) socioeconomic status. Body mass index (body weight/height^2^; kg/m^2^) was measured according to international standardization ^25^.

The sample size estimation was performed a priori, and more information is available in a previous publication ^19^ ^,^ ^26^. The final sample of 1,085 students holds statistical power (β = 0.80 and 5% significance level for two-tailed tests) to detect small mediation effects (standardized change of 0.14 or greater) when testing mediation of the effect of intervention in physical activity, even for subgroups analyses ^27^.

Baseline characteristics were described by means (standard deviation - SD) and frequencies for continuous and categorical variables, respectively. Log-transformation was performed for moderate-to-vigorous physical activity after normality distribution was violated by a visual inspection and skewness/kurtosis and Kolmogorov-Smirnov tests. Differences at baseline between control and intervention groups were evaluated using the chi-squared test (categorical variables) and linear mixed models (continuous variables), adjusted for school-level clustering by including a random intercept for schools.

Mediation analysis was performed using the SPSS PROCESS macro, version 2.12 (http://www.processmacro.org/index.html) ^28^. The product of ab coefficient was performed ^29^. The following were analyzed (coefficient β): the total effect of the intervention on moderate-to-vigorous physical activity (coefficient *c*); the effect of the intervention on potential mediators (coefficient *a*); association between the potential mediator and moderate-to-vigorous physical activity (coefficient *b*). The product of *ab* coefficients and their standardized error (500 bootstrapping resampling) were estimated ^30^ (Supplementary Material - Figure S1; https://cadernos.ensp.fiocruz.br/static//arquivo/supl-e00138023_3745.pdf). Mediation was confirmed if the product of coefficients was statistically significant ^15^. Non-standardized scores and mediated proportion (indirect effect by total effect rate) were estimated ^15^. Simple and multiple (including variables with statistical significance in the simple model) mediation models were performed ^29^. Analyses were performed for the total sample and by sex, adjusted for baseline variables (coefficient a: mediators, moderate-to-vigorous physical activity, age, body mass index, socioeconomic level, and school; coefficient b: mediators, treatment, age, body mass index, and socioeconomic level). The significance level was set at 5% for two-tailed tests.

## Results

A total of 1,085 students enrolled in the Strengthen Your Health program (intervention group n = 548), and most were boys (51.5%). Table 1 displays the characteristics of the intervention and control groups for boys and girls. Most boys were 14-18 years old (51.9%), while most girls were 11-13 years old (58%). Most boys and girls were from low socioeconomic status (70.6% and 78.3%, respectively) and had healthy nutritional status (76.4% and 73%, respectively). Random intercept models revealed that the proportion of variance explained by the differences between schools was negligible, with ICC ranging from < 0.001 (for moderate-to-vigorous physical activity in both boys and girls) to 0.05 (perception of school environment in boys).

**Table 1:** Students’ characteristics at baseline according to group allocation and status participation from the Strengthen Your Health program. Brazil, 2014.

Characteristic	Boys			Girls		
	Intervention (n = 284)	Control (n = 275)	p-value	Intervention (n = 264)	Control (n = 262)	p-value
	Mean (SD)	Mean (SD)		Mean (SD)	Mean (SD)	
Age group (11-13 years) *	49.3 (140)	46.9 (129)	0.572	59.5 (157)	56.5 (148)	0.489
Socioeconomic status (lower) *	69.9 (197)	71.3 (194)	0.705	75.3 (198)	81.3 (213)	0.095
Overweight *	26.4 (75)	20.7 (57)	0.114	30.3 (80)	23.7 (62)	0.086
Moderate-to-vigorous physical activity (minutes/week)	670.74 (717.28)	751.97 (805.56)	0.029	349.72 (420.756)	344.46 (451.64)	0.628
Attitude	16.23 (2.35)	16.40 (2.44)	0.420	15.64 (2.43)	15.43 (2.68)	0.347
Self-efficacy	21.08 (3.76)	21.01 (3.60)	0.813	20.38 (3.23)	20.56 (3.38)	0.538
Social support from friends	12.88 (4.45)	13.29 (4.72)	0.291	9.70 (3.71)	9.70 (4.18)	0.822
Social support from parents	11.71 (4.43)	11.31 (4.26)	0.283	10.61 (4.16)	10.27 (4.00)	0.337
Social support from teachers	9.97 (3.57)	11.08 (4.00)	0.001	10.88 (4.29)	10.97 (4.13)	0.642
Perception of neighborhood safety	12.62 (2.52)	11.95 (3.15)	0.209	12.56 (2.65)	11.72 (2.70)	0.073
Perception of neighborhood facilities	13.06 (2.88)	12.27 (2.76)	0.001	12.13 (2.81)	11.60 (2.79)	0.079
Perception of school environment	7.48 (1.79)	6.89 (1.80)	0.129	7.23 (1.75)	6.65 (1.80)	0.187

SD: standard deviation.

Note: p-value for continuous variables: linear mixed models, adjusted for school-level clustering as a random effect; values written in bold: statistical significance (p < 0.05).

* Values displayed were represented as percentage and frequencies: % (n).

Table 2 shows the results from simple mediation analysis. Table 3 shows the results from multiple mediation analyses in the total sample. We found that attitude towards physical activity practice (mediation: 13.8%), social support from friends (mediation: 15%), and social support from teachers (mediation: 8.6%) were statistically significant mediators, explaining a total of 40% of the effect of the Strengthen Your Health program on moderate-to-vigorous physical activity.

**Table 2:** Multiple mediation analysis of the effect of the Strengthen Your Health program on the moderate-to-vigorous physical activity in the total sample. Brazil, 2014.

Potential mediators	**Coefficient *a* (95%CI)**	p-value	**Coefficient *b* (95%CI)**	p-value	Coefficient *ab* (95%CI) *	p-value	Mediation (%)
Total effect (*c*)					0.706 (0.276; 1.136)	< 0.01	
Direct effect (*c’*)					0.423 (-0.003; 0.849)	0.05	
Attitude	1.108 (0.488; 1.728)	< 0.01	0.088 (0.046; 0.130)	< 0.01	0.097 (0038; 0.192)		13.8
Social support from friends	1.535 (0.550; 2.521)	< 0.01	0.069 (0.042; 0.096)	< 0.01	0.106 (0.038; 0.201)		15.0
Social support from teachers	2.140 (1.144; 3.137)	< 0.01	0.028 (0.002; 0.055)	0.03	0.061 (0.006; 0.140)		8.6
Perception of school environment	0.967 (0.469; 1.465)	< 0.01	0.019 (-0.033; 0.071)	0.47	0.019 (-0.026; 0.073)		2.6
Multiple mediation analysis					0.282 (0.154; 0.444)		40.0

95%CI: 95% confidence interval.

Note: values written in bold: statistical significance (p < 0.05); coefficient *a*: non-standardized coefficient of the effect of treatment (intervention vs. control) on the potential mediator, adjusted for baseline variables (mediators, moderated-to-vigorous physical activity, age, body mass index, socioeconomic level, and school); coefficient *b*: non-standardized coefficient of the association between potential mediator and moderate-to-vigorous physical activity, adjusted for baseline variables (mediators, treatment, age, body mass index, and socioeconomic level); coefficient *c*: non-standardized coefficient of the total effect of treatment (intervention vs. control) on moderate-to-vigorous physical activity, adjusted for baseline cofounder values; coefficient *c’*: the direct effect of the intervention on moderate-to-vigorous physical activity; coefficient *ab*: product of coefficients *a* and *b*;

* 95%CI confidence interval based on bootstrapping (5,000 samples).

**Table 3:** Multiple mediation analysis of the effect of the Strengthen Your Health program on the moderate-to-vigorous physical activity in students with data imputation. Brazil, 2014.

Potential mediators	**Coefficient *a*** **(95%CI)**	p-value	**Coefficient *b*** **(95%CI)**	p-value	**Coefficient *ab*** **(95%CI)**	p-value	Mediation (%)
Boys (n = 609)							
Total effect					0.549 (0.072; 1.026)	0.024	
Direct effect					0.267 (-0.201; 0.735)	0.260	
Attitude	1.360 (0.584; 2.136)	< 0.010	0.107 (0.058; 0.156)	< 0.010	0.145 (0.046; 0.304)		26.4
Social support friends	1.838 (0.534; 3.143)	0.017	0.058 (0.028; 0.087)	< 0.010	0.106 (0.022; 0.215)		19.3
Social support teachers	1.500 (0.273; 2.727)	< 0.010	0.021 (-0.010; 0.052)	0.177	0.032 (-0.020; 0.099)		5.8
Multiple mediation analysis					0.282 (0.131; 0.456)		51.4
Girls (n = 573)							
Total effect					1.025 (0.403; 1.647)	< 0.010	
Direct effect					0.763 (0.132; 1.394)	0.020	
Attitude	0.925 (0.102; 1.747)	0.028	0.092 (0.029; 0.154)	0.010	0.085 (0.004; 0.209)		8.3
Social support teachers	3.026 (1.720; 4.332)	< 0.010	0.046 (0.006; 0.085)	0.020	0.138 (0.015; 0.302)		13.5
Perception of school environment	1.006 (0.374; 1.637)	< 0.010	0.038 (-0.043; 0.120)	0.360	0.038 (-0.035; 0.129)		3.8
Multiple mediation analysis					0.262 (0.091; 0.482)		25.5

95%CI: 95% confidence interval.

Note: values written in bold: statistical significance (p < 0.05); coefficient *a*: non-standardized coefficient of the effect of treatment (intervention vs. control) on the potential mediator; coefficient *b*: non-standardized coefficient of the association between potential mediator and moderate-to-vigorous physical activity; coefficient *c*: non-standardized coefficient of the total effect of treatment (intervention vs. control) on moderate-to-vigorous physical activity; coefficient *ab*: product of coefficients *a* and *b*.

Table 4 presents the results from multiple mediation analyses according to sex. Among boys, attitude and social support from friends showed statistically significant *ab* coefficients, indicating a statistically significant indirect effect of the program on moderate-to-vigorous physical activity via these mediators, showing mediation values of 25.5% and 20.2%, respectively. The significant mediator role of social support from teachers in simple mediation analysis was no longer significant in the multiple mediation model. Among girls, attitude and social support from teachers were mediators, showing values of 8% and 13.6%, respectively. The perception of the school environment was no longer a statistically significant mediator when included in the multiple mediation model. Analyzes with imputed data showed similar results and can be verified in the Supplementary Material (Tables S2 and S3; https://cadernos.ensp.fiocruz.br/static//arquivo/supl-e00138023_3745.pdf).

**Table 4:** Multiple mediation analysis of the effect of the Strengthen Your Health program on the moderate-to-vigorous physical activity in students. Brazil, 2014.

Potential mediator	**Coefficient *a* (95%CI)**	p-value	**Coefficient *b* (95%CI)**	p-value	**Coefficient *ab* (95%CI)**	p-value	Mediation (%)
Boys (n = 559)							
Total effect (*c*)					0.557 (0.200; 1.095)	0.040	
Direct effect (*c’*)					0.255 (-0.251; 0.801)	0.300	
Attitude	1.291 (0.429; 2.152)	< 0.010	0.110 (0.056; 0.164)	< 0.010	0.142 (0.046; 0.304)		25.5
Social support from friends	0.197 (0.512; 3.436)	0.010	0.057 (0.025; 0.089)	< 0.010	0.113 (0.027; 0.256)		20.2
Social support from teachers	1.48 (0.081; 2.872)	0.040	0.019 (-0.014; 0.051)	0.270	0.027 (-0.014; 0.128)		4.90
Multiple mediation analysis					0.282 (0.132; 0.506)		50.6
Girls (n = 526)							
Total effect (*c*)					0.992 (0.307; 1.677)	< 0.010	
Direct effect (*c’*)					0.747 (0.052; 1.442)	0.030	
Attitude	0.906 (0.001; 1.809)	0.050	0.088 (0.214; 0.154)	0.010	0.079 (0.006; 0.227)		8.00
Social support from teachers	3.012 (1.589; 4.435)	< 0.010	0.045 (0.003; 0.087)	0.040	0.135 (0.019; 0.328)		13.6
Perception of school environment	1.027 (0.341; 1.713)	< 0.010	0.030 (-0.057; 0.118)	0.500	0.031 (-0.040; 0.141)		3.10
Multiple mediation analysis					0.245 (0.073; 0.492)		24.7

95%CI: 95% confidence interval.

Note: values written in bold: statistical significance (p < 0.05); coefficient *a*: non-standardized coefficient of the effect of treatment (intervention vs. control) on the potential mediator, adjusted for baseline variables (mediators, moderated-to-vigorous physical activity, age, body mass index, socioeconomic level, and school); coefficient *b*: non-standardized coefficient of the association between potential mediator and moderate-to-vigorous physical activity, adjusted for baseline variables (mediators, treatment, age, body mass index, and socioeconomic level); coefficient *c*: non-standardized coefficient of the total effect of treatment (intervention vs. control) on moderate-to-vigorous physical activity, adjusted for baseline cofounder values; coefficient *c’*: the diretct effect of the intervention on moderate-to-vigorous physical activity; coefficient *ab*: product of coefficients *a* and *b*.

## Discussion

We found that variables from different levels of the socioecological model (e.g., attitude, social support from friends, and social support from teachers) were positively impacted by the program and were mediators of the effect of the Strengthen Your Health program on moderate-to-vigorous physical activity. However, there was a difference between boys and girls on how social support helps explain the changes in moderate-to-vigorous physical activity. Support from friends was significantly relevant for boys, whereas social support from teachers was significant for girls.

Attitude was found to be a mediator that partially explained the effects of the intervention on moderate-to-vigorous physical activity in both boys and girls, revealing a central role as a determinant of physical activity. These findings corroborate a recent systematic review that argues that interventions aimed at promoting physical activity should facilitate positive affective variables (such as attitude) to increase physical activity engagement in adolescents ^12^. In short, boys and girls will be more likely to engage in physical activity behavior if they improve their attitude towards physical activity since there is an improvement in the perception of physical activity importance, enjoyment, safety, and fun during physical activity practice. Providing positive experiences and strengthening familiarity with physical activity can also increase physical literacy levels, a concept that postulates that increasing affective (motivation and confidence), physical (physical confidence), and cognitive (knowledge and understanding) aspects of physivcal activity can increase the value and responsibility for getting engaged in physical activity. Better activities lead to greater chances of engaging in long-term physical activity behavior ^31^.

Strategies from the Strengthen Your Health program were designed to increase these attitude-related elements, and future interventions may benefit from these findings, as most interventions failed to improve attitudes toward physical activity in adolescents ^11^. Specifically, a previous study showed that the Strengthen Your Health program showed an effect size of 0.29 on attitude. The intervention offered several activities to develop students’ knowledge and interest, which may have helped increase the attitude toward physical activity ^21^. In addition to being one of the mediators that most explained physical activity change, attitude contributed to physical activity change in both boys and girls, further strengthening its importance and contributing to the advancement of the field, as its sex-specific role has not yet been fully elucidated ^12^.

Social support mediated the effect of the intervention on moderate-to-vigorous physical activity, so the greater the perception of social support from teachers and friends, the more significant the changes in moderate-to-vigorous physical activity. Understanding the social support physical activity association is a complex topic, as it can present different patterns according to type and providers of social support, type of physical activity, and vary according to sex ^32^. In fact, this study found that most but not all providers of social support were able to change moderate-to-vigorous physical activity behavior; moreover, it appears that providers of social support may impact girls and boys differently.

Social support from friends was found to be an important mediator of the effect of the intervention on moderate-to-vigorous physical activity. Moreover, a previous study of the Strengthen Your Health program showed a 0.24 effect size on social support from friends ^21^. Thus, it means that offering activities that promote interaction among adolescents, such as encouraging them to talk and stimulating them to play with peers, seems to be an effective strategy for promoting moderate-to-vigorous physical activity. In line with this, it was previously suggested that physical activity could be promoted via peer-led strategies, and social support techniques can be a mechanism for a successful strategy, being a critical factor in adopting and maintaining physical activity behavior in adolescents ^33^.

Teachers play a central role in promoting physical activity. Stimulating, talking, and commenting on physical activity in the classroom can be an effective way for teachers to promote physical activity, even in classes other than Physical Education. Encouraging teachers to includephysical activity related topics in their classrooms can increase students’ perception of social support, which can positively impact physical activity. The effect size of the Strengthen Your Health program on social support from teachers was 0.34 ^21^. These results confirm that, when encouraged and provided with the means to do so, teachers can play a positive role in physical activity behavior in adolescents ^32^. These findings corroborate the Fit-4-Fun Program, in which the increase in physical activity behavior in adolescents was mainly due to increased social support from teachers ^34^. An interesting observation from both programs is that teachers should provide support during their regular working hours rather than spending extra time from their work shifts. The potential of teacher training should be further considered and studied to overcome the topic that has so far been poorly understood ^35^.

The fact that sex-specific analysis revealed that providers of social support might impact physical activity differently among boys and girls is not surprising. Previous studies have highlighted the importance of considering these differences ^32^. Thus, the mechanisms that could explain these findings are yet to be elucidated. It is hypothesized that the girls perceived more social support from teachers because some strategies of the Strengthen Your Health program were girls-target physical activity opportunities, such as the Gym in Schools, with physical activity breaks in the school recess, including exercises, dances, and music-based games. Moreover, it is also possible that boys were more engaged in group-based physical activity, which increased their awareness of social support from their peers.

The Strengthen Your Health program positively impacted students’ perception of the school environment, especially among girls; however, it was not related to changes on moderate-to-vigorous physical activity or was not a mediator of the program. This variable was evaluated considering only the perception of sports material availability and the presence and quality of places to practice physical activity. The environment is a complex topic that can be assessed objectively and subjectively and can comprise different dimensions, such as social, physical, and political ^36^. In that way, it is not possible to rule out the potential role of the environment on physical activity, which has been previously evidenced ^37^.

Finally, self-efficacy and social support from parents were not confirmed as mediators. Moreover, the intervention was not able to impact these variables, as shown by the results of the simple mediation analysis. Some arguments may explain these unexpected findings. In fact, a more in-depth analysis of the implementation program found that parents were not aware of the Strengthen Your Health program intervention, which reveals the failure of the strategy of reaching parents by sending them flyers with health-related information ^22^. Moreover, the fact that most families in the study had a lower socioeconomic status, combined with the context of a low HDI, can further amplify the challenges in implementing the program ^19^, especially the implementation of health education strategies - the low engagement of parents in school-based interventions has also been observed in the literature from high-income countries ^38^.

Some strengths of this study should be mentioned. This is one of the first interventions conducted in a low HDI area from low- and middle-income countries, a highly vulnerable region. These findings may pave the way for future physical activity interventions in low HDI areas, as they share some common contextual factors and similar physical activity patterns. For example, in high HDI countries, some subgroups in the population, including women, presented better physical activity indicators when compared with lower HDI countries. Moreover, high HDI countries may offer better sources of influence on community, environment, and government indicators compared with low HDI countries ^39^. This study is also one of the first conducted in the Brazilian context, aimed at identifying mediators of a school-based program to promote physical activity. Full-time schools have increased since 2014 (the year of the program) in Brazil, especially as part of regional and national policies that aim to promote education and health in vulnerable areas. Thus, our results may be considered to improve current and future policies based on healthy schools. The study considered the different levels of factors that may influence physical activity behavior from a socioecological perspective, assessed by validated instruments with statistically adequate sample size, and analyzed by robust mediation methods. Nevertheless, some limitations must also be mentioned. Self-assessment was applied to measure physical activity, which can be impacted by memory bias and social desirability. However, the instrument was previously validated ^23^; the aim was to compare the pre-post effect between groups, and the same instrument was applied in control and intervention schools. The researchers were not blinded to the control and intervention groups. However, they were trained to follow a standardized protocol in both control and intervention schools. The intervention lasted four months, and only the immediate post-intervention was evaluated with no follow-up, not allowing an assessment of the long-term effect of the intervention on physical activity related outcomes. Thus, it was not possible to infer the causality between mediators and physical activity because they were assessed at the same time point. Complete data were performed instead of intention-to-treat analysis ^40^. However, the study presented only 8.2% missing data (n = 97 dropouts), and participants and dropouts were similar in all variables (except that dropouts were slightly older than participants). Finally, the sampling design was not specifically delineated to detect statistical differences between sexes. Future interventions should incorporate these aspects from the inception of the program to actively contribute to mitigating inequality in population segments.

## Conclusion

Our findings highlight that a school-based intervention that goes beyond the individual level and expands to the social aspects of the school environment can positively impact physical activity behavior and its psychosocial factors. In particular, a relevant part of physical activity behavior change was derived from the improvement of psychosocial factors; the mediation effects (% of explanation) were around 50% for boys and 25% for girls. Attitude towards physical activity was a relevant mediator for boys and girls. However, sex-specific patterns were identified, as social support appeared to play a distinct role as a mediator of the physical activity; support from friends being relevant for boys and social support from teachers for girls. The findings reinforce the school’s potential as a place to promote physical activity, but it is important to consider that there is no one-size-fits-all approach to account for all peculiarities among students. More research evaluating this evidence will aid schools reach their full potential as enabling settings that encourage adolescents to incorporate physical activity behavior into their lifestyles.

### Perspective

To date, knowledge on how to improve physical activity among students from vulnerable areas is scarce. The findings of this study pointed that focusing not only on individual aspects but also on the social aspects of the school environment can aid to successfully improve physical activity. This can be outlined by school-based interventions that focus on the curriculum, changes in the school environmental and educational strategies, and the family and school community. More studies are needed to confirm the findings, contributing to impact public health policies.

## References

[1] World Health Organization (2018). Global action plan on physical activity 2018-2030: more active people
for a healthier world..

[2] World Health Organization (2021). Promoting physical activity through schools: a toolkit..

[3] Ramires V, Santos P, Barbosa V, Bandeira A, Tenório M, Camargo E (2023). Physical education for health among school-aged children and
adolescents: a scoping review of reviews.. J Phys Act Health.

[4] Rhodes RE, McEwan D, Rebar AL (2019). Theories of physical activity behaviour change: a history and
synthesis of approaches.. Psychol Sport Exerc.

[5] Stokols D (1992). Establishing and maintaining healthy environments. Toward a
social ecology of health promotion.. Am Psychol.

[6] Neil-Sztramko SE, Caldwell H, Dobbins M (2013). School-based physical activity programs for promoting physical
activity and fitness in children and adolescents aged 6 to
18.. Cochrane Database Syst Rev.

[7] Love R, Adams J, Sluijs EMF (2019). Are school-based physical activity interventions effective and
equitable? A meta-analysis of cluster randomized controlled trials with
accelerometer-assessed activity.. Obes Rev.

[8] Barbosa VC, Minatto G, Mota J, Silva KS, Campos W, Lopes AS (2016). Promoting physical activity for children and adolescents in low-
and middle-income countries: an umbrella systematic review.. Prev Med.

[9] United Nations Development Programme (2014). Human Development Report 2014. Sustaining human progress: reducing
vulnerabilities and building resilience..

[10] Silva DAS (2015). Relationship between Brazilian adolescents&apos; physical
activity and social and economic indicators of the cities where they
live.. Percept Mot Skills.

[11] van Stralen MM, Yildirim M, te Velde SJ, Brug J, van Mechelen W, Chinapaw MJ (2011). What works in school-based energy balance behaviour interventions
and what does not? A systematic review of mediating
mechanisms.. Int J Obes (Lond).

[12] Chen C, Finne E, Kopp A, Jekauc D (2020). Can positive affective variables mediate intervention effects on
physical activity? A systematic review and meta-analysis.. Front Psychol.

[13] Lubans DR, Foster C, Biddle SJH (2008). A review of mediators of behavior in interventions to promote
physical activity among children and adolescents.. Prev Med.

[14] Cain MK, Zhang Z, Bergeman CS (2018). Time and other considerations in mediation
design.. Educ Psychol Meas.

[15] Cerin E (2010). Ways of unraveling how and why physical activity influences
mental health through statistical mediation analyses.. Ment Health Phys Act.

[16] Guthold R, Stevens GA, Riley LM, Bull FC (2020). Global trends in insufficient physical activity among
adolescents: a pooled analysis of 298 population-based surveys with 1.6
million participants.. Lancet Child Adolesc Health.

[17] Bull FC, Al-Ansari SS, Biddle S, Borodulin K, Buman MP, Cardon G (2020). World Health Organization 2020 guidelines on physical activity
and sedentary behaviour.. Br J Sports Med.

[18] Moher D, Hopewell S, Schulz KF, Montori V, Gøtzsche PC, Devereaux PJ (2012). CONSORT 2010 explanation and elaboration: updated guidelines for
reporting parallel group randomised trials.. Int J Surg.

[19] Barbosa VC, Lopes AS, Lima AB, Souza EA, Gubert FA, Silva KS (2015). Rationale and methods of a cluster-randomized controlled trial to
promote active and healthy lifestyles among Brazilian students: the
“Fortaleça sua Saúde” program.. BMC Public Health.

[20] Barbosa VC, Silva KS, Mota J, Beck C, Silva Lopes A (2016). A physical activity intervention for Brazilian students from low
human development index areas: a cluster-randomized controlled
trial.. J Phys Act Health.

[21] Barbosa VC, Silva KS, Mota J, Vieira NFC, Gubert FA, Lopes AS (2017). “For whom was it effective?” Moderators of the effect of a
school-based intervention on potential physical activity determinants among
Brazilian students.. Prev Med.

[22] Lopes IE, Linard JG, Silva ML, Barbosa VC (2020). Implementação do programa de promoção do estilo de vida ativo em
estudantes: o “Fortaleça sua Saúde”.. J Phys Educ (Maringá).

[23] Farias JC, Lopes AS, Mota J, Santos MP, Ribeiro JC, Hallal PC (2012). Validade e reprodutibilidade de um questionário para medida de
atividade física em adolescentes: uma adaptação do Self-Administered
Physical Activity Checklist.. Rev Bras Epidemiol.

[24] Associação Brasileira de Empresas de Pesquisa Critério de classificação econômica Brasil..

[25] Lohman TG, Roche AF, Martorell R (1988). Anthropometric standardization reference manual..

[26] Nahas MV, Barros MVG, Assis MAA, Hallal PC, Florindo AA, Konrad L (2009). Methods and participant characteristics of a randomized
intervention to promote physical activity and healthy eating among brazilian
high school students: the Saude na Boa project.. J Phys Act Health.

[27] Pan H, Liu S, Miao D, Yuan Y (2018). Sample size determination for mediation analysis of longitudinal
data.. BMC Med Res Methodol.

[28] Hayes AF (2022). Introduction to mediation, moderation, and conditional process analysis:
a regression-based approach..

[29] MacKinnon DP, Fairchild AJ, Fritz MS (2007). Mediation analysis.. Annu Rev Psychol.

[30] Hayes AF (2009). Beyond Baron and Kenny: statistical mediation analysis in the new
millennium.. Communication Monographs.

[31] Carl J, Barratt J, Töpfer C, Cairney J, Pfeifer K (2022). How are physical literacy interventions conceptualized? A
systematic review on intervention design and content.. Psychol Sport Exerc.

[32] Laird Y, Fawkner S, Kelly P, McNamee L, Niven A (2016). The role of social support on physical activity behaviour in
adolescent girls: a systematic review and meta-analysis.. Int J Behav Nutr Phys Act.

[33] McHale F, Ng K, Taylor S, Bengoechea E, Norton C, O&apos;Shea D (2022). A systematic literature review of peer-led strategies for
promoting physical activity levels of adolescents.. Health Educ Behav.

[34] Eather N, Morgan PJ, Lubans DR (2013). Social support from teachers mediates physical activity behavior
change in children participating in the Fit-4-Fun
intervention.. Int J Behav Nutr Phys Act.

[35] Lander N, Eather N, Morgan PJ, Salmon J, Barnett LM (2017). Characteristics of teacher training in school-based physical
education interventions to improve fundamental movement skills and/or
physical activity: a systematic review.. Sports Med.

[36] Turner K, Foster C, Allender S, Plugge E (2015). A systematic review of how researchers characterize the school
environment in determining its effect on student obesity.. BMC Obes.

[37] Silva JA, Duca GFD, Lopes MVV, Knebel MTG, Streb AR, Matias TS (2023). Patterns of school environment that matter for physical activity
engagement among Brazilian adolescents.. Sport Sciences for Health.

[38] Verjans-Janssen SRB, van de Kolk I, Van Kann DHH, Kremers SPJ, Gerards SMPL (2018). Effectiveness of school-based physical activity and nutrition
interventions with direct parental involvement on children&apos;s BMI
and energy balance-related behaviors - a systematic review.. PLoS One.

[39] Silva DAS, Aubert S, Ng K, Morrison SA, Cagas JY, Tesler R (2022). Association between physical activity indicators and Human
Development Index at a national level: information from Global Matrix 4.0
Physical Activity Report Cards for Children and Adolescents.. J Phys Act Health.

[40] Cheng ST, Chan WC, Fung HH, Lam LCW (2022). Self-efficacy in controlling upsetting thoughts, but not positive
gains, mediates the effects of benefit-finding group intervention for
Alzheimer family caregivers.. Psychol Aging.

