# Supplementary material for: Pathways of physical activity behavior after an intervention with students from vulnerable areas: a cluster randomized controlled trial based on a socioecological approach
Source: Cad Saude Publica. 2024 Nov 11;40(9):e00138023. doi: 10.1590/0102-311XEN138023 (PMC11560138; doi:10.1590/0102-311XEN138023)
Supplement: Supplementary file 1 [file 1678-4464-csp-40-09-EN138023-s.pdf]

Table S1 Intraclass correlation coefficient (ICC) of scales of intrapersonal, interpersonal, and environmental items.

Item	ICC
**Attitude toward physical activity**	0.62
Unimportant/Important	0.72
Unsafe/Safe	0.63
Bad/Good	0.58
Harmful/Healthy	0.65
Boring/Fun	0.59
**Self-efficacy**	0.70
Lack of company	0.67
Demotivated	0.66
Screen access in free-time	0.62
Invitation to do other things by friends	0.64
Laziness	0.54
Lack of physical activity skills	0.70
Lack of places near home	0.68
Lack of instruction	0.68
**Social support from friends**	0.66
Encouragement	0.61
Physical activity practice together	0.67
Invitation to physical activity with them	0.63
Observation (watching)	0.71
Positive comments	0.63
**Social support from parents**	0.62
Encouragement	0.66
Physical activity practice together	0.64
Providing transport to physical activity	0.62
Observation (watching)	0.59
Positive comments	0.56
Conversation (talking)	0.63
**Social support from teachers**	0.69
Encouragement	0.70
Invitation to physical activity with them	0.66
Supervision (watching)	0.64
Positive comments	0.67
Conversation (talking)	0.61
**Perception of neighborhood safety**	0.58
Pollution	0.64
Condition of sidewalks	0.64
Safety for walking	0.62
General safety	0.57
Safety for cycling	0.58
**Perception of neighborhood facilities**	0.67
Physical activity facilities	0.65
Physical activity practice by peers	0.63
Physical activity places	0.61
Proximity of physical activity places	0.85
General appearance	0.64
**Perception of school environment**	0.65
Physical activity facilities in free-time	0.58
Enjoyable physical activity facilities	0.63
Appearance of physical activity facilities	0.66

Note: adapted from Barbosa Filho et al (2016).

Table S2 Multiple mediation analysis of the effect of the Strengthen Your Health program on the moderate-to-vigorous physical activity in the total sample with imputation data. Brazil, 2014.

**Potential mediators**	**Coefficient *a* (95%CI)**	**p-value**	**Coefficient *b* (95%CI)**	**p-value**	**Coefficient *ab* (95%CI) ***	**p-value**	**Mediation (%)**
Total effect					**0.750 (0.362; 1.139)**	**< 0.010**	
Direct effect					**0.446 (0.062; 0.830)**	**0.023**	
Attitude	**1.133 (0.571; 1.696)**	**< 0.010**	**0.090 (0.051; 0.130)**	**< 0.010**	**0.103 (0.040; 0.180)**		**13.7**
Social support friends	**1.668 (0.763; 2.571)**	**< 0.010**	**0.074 (0.049; 0.099)**	**< 0.010**	**0.123 (0.050; 0.211)**		**16.4**
Social support teachers	**2.184 (1.284; 3.084)**	**< 0.010**	**0.025 (0.000; 0.049)**	**0.049**	0.054 (-0.001; 0.117)		7.1
Perception of school environment	**0.934 (0.480; 1.387)**	**< 0.010**	0.027 (-0.021; 0.075)	0.276	0.025 (-0.019; 0.076)		3.3
Multiple mediation analysis					**0.304 (0.175; 0.447)**		**40.5**

95%CI: 95% confidence interval.

Note: values written in bold: statistical significance ($p < 0.05$); coefficient *a*: non-standardized coefficient of the effect of treatment (intervention vs. control) on the potential mediator; coefficient *b*: non-standardized coefficient of the association between potential mediator and moderate-to-vigorous physical activity; coeffcient *c*: non-standardized coefficient of the total effect of treatment (intervention vs. control) on moderate-to-vigorous physical activity; coefficient *ab*: product of coefficients a and b.

* 95%CI confidence interval based on bootstraping (5,000 samples).

Table S3 Multiple mediation analysis of the effect of the Strengthen Your Health program on the moderate-to-vigorous physical activity in girls and boys students with imputation data. Brazil, 2014.

**Potential mediators**	**Coefficient *a* (95%CI)**	**p-value**	**Coefficient *b* (95%CI)**	**p-value**	**Coefficient *ab* (95%CI)**	**p-value**	**Mediation (%)**
**Boys (n = 609)**							
Total effect					0.549 (0.072; 1.026)	0.024	
Direct effect					0.267 (-0.201; 0.735)	0.260	
Atittude	1.360 (0.584; 2.136)	< 0.010	0.107 (0.058; 0.156)	< 0.010	0.145 (0.046; 0.304)		26.40
Social support friends	1.838 (0.534; 3.143)	0.017	0.058 (0.028; 0.087)	< 0.010	0.106 (0.022; 0.215)		19.30
Social support teachers	1.500 (0.273; 2.727)	< 0.010	0.021 (-0.010; 0.052)	0.177	0.032 (-0.020; 0.099)		5.80
Multiple mediation analysis					0.282 (0.131; 0.456)		51.40
**Girls (n = 573)**							
Total effect					1.025 (0.403; 1.647)	< 0.010	
Direct effect					0.763 (0.132; 1.394)	0.020	
Atittude	0.925 (0.102; 1.747)	0.028	0.092 (0.029; 0.154)	0.010	0.085 (0.004; 0.209)		8.30
Social support teachers	3.026 (1.720; 4.332)	< 0.010	0.046 (0.006; 0.085)	0.020	0.138 (0.015; 0.302)		13.50
Perception of school environment	1.006 (0.374; 1.637)	< 0.010	0.038 (-0.043; 0.120)	0.360	0.038 (-0.035; 0.129)		3.80
Multiple mediation analysis					0.262 (0.091; 0.482)		25.50

95%CI: 95% confidence interval.

Notes: values written in bold: statistical significance ($p < 0.05$); coefficient *a*: non-standardized coefficient of the effect of treatment (intervention vs. control) on the potential mediator; coefficient *b*: non-standardized coefficient of the association between potential mediator and moderate-to-vigorous physical activity; coeffcient *c*: non-standardized coefficient of the total effect of treatment (intervention vs. control) on moderate-to-vigorous physical activity; coefficient *ab*: product of coefficients *a* and *b*; 95%CI confidence interval based on bootstraping (5,000 samples).

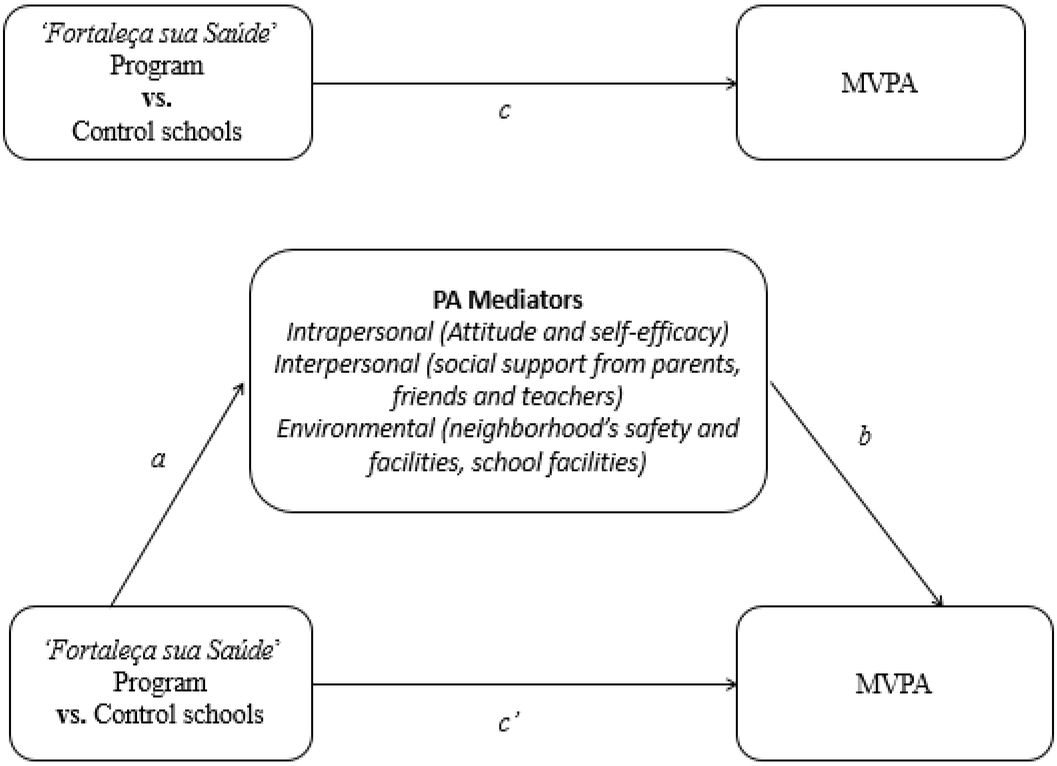

Figure S1 Theoretical model of the Strengthen Your Health program [*Fortaleça Sua Saúde* program].

MVPA: moderate-to-vigorous physical activity; PA: physical activity.
Note: coefficient *a*: intervention effect on potential mediators; coefficient *b*: association between potential mediators and moderate-to-vigorous physical activity; coefficient *c*: the total effect of the intervention on moderate-to-vigorous physical activity, coefficient *c'*: the direct effect of the intervention on moderate-to-vigorous physical activity.
